# Influence of microwave drying on quality parameters of foamed Nagpur Mandarin *(Citrus reticulata)* juice

**DOI:** 10.1016/j.heliyon.2024.e30449

**Published:** 2024-04-27

**Authors:** Bhagyashree Nivrutti Patil, Suchita V. Gupta, Nivrutti.B. Patil, Nileshwari Yewle

**Affiliations:** a Department of Agricultural Process Engineering, Dr. Panjabrao Deshmukh Krishi Vidyapeeth, Akola, Maharashtra, India; b Krishi Krishi Vigyan Kendra Karda, Washim, Maharashtra, India; c Department of Botany and Plant Pathology, Purdue University, West Lafayette, USA

**Keywords:** Nagpur Mandarin powder, Microwave power, Foaming agent, Drying bed thickness

## Abstract

In the world of industrial drying processes, foam mat microwave drying is a significant and valuable approach. Its advantages include increased drying effectiveness, preservation of product quality, energy and cost savings, flexibility in application, and improved safety. Development of Nagpur mandarin juice powder is tedious and time-consuming due to its bitter test and less total soluble solid, therefore the present research carried out with the process parameters for microwave drying include microwave power levels (180, 360, 540, 720, and 900 W) and drying bed thicknesses (2, 4, and 6 mm). Foamed juice is produced using soy protein isolate (2.10 %), GMS (2.75 %), CMC (1.75 %), and sugar (5.10 %), with whipping times of 8 min. Additional foaming agents include guar gum (0.45 %), soy protein isolate (3.30 %), and sugar (10 %) with whipping times of 6 min. The optimal conditions for drying Nagpur mandarin juice were determined through analysis using Design-Expert 11.0.4.1 software. These conditions include 540 W of microwave power, a drying bed thickness of 3 mm, and the use of a foaming agent comprising 2.10 % soy protein isolate, 2.75 % GMS, 1.75 % CMC, and 5.10 % sugar, with an 8-min whipping period. Under these optimized conditions, the resulting powder exhibited the following characteristics: color b value of 19.59, ΔE (change in color) of 6.24, acidity of 0.40 %, ascorbic acid content of 36.64 mg/100 g, water activity of 0.26, drying time (in minutes), and overall acceptability rating of 7.77. These findings highlight the effectiveness of the optimized process parameters in achieving desirable quality attributes for Nagpur mandarin juice powder production.

## 1 Introduction

Nagpur Mandarin (Citrus reticulata) is a renowned citrus variety from India, known for its vibrant color, sweet-tangy flavor, and juicy pulp. It is one of the most important commercial citrus species in India, along with sweet orange (Citrus sinensis) and acid lime (Citrus aurantifolia), contributing 41 %, 25 %, and 23 % respectively to the country's total citrus production [1]. India stands third in global citrus production at 12.74 million tonnes, with an area of 10.55 million hectares and productivity of 10.4 MT/ha, while the total orange production in India is 3431.4 thousand MT (3.9 % of total fruit production) from an area of 330.0 thousand hectares, with a productivity of 10.4 MT/ha [2]. Citrus fruits are valued not only for their ascorbic acid (Vitamin C) content but also for providing essential nutrients like glycemic and non-glycemic carbohydrates, potassium, calcium, vitamins, and phytochemicals [3,4].

Dehydrated foods require retaining key quality attributes like nutritional content, flavor, aroma, color, and storage stability. Drying fruits and vegetables enhances shelf-life, minimizes packaging needs, and reduces transportation weight [5]. However, drying sugar-rich foods like fruit pulps and juices into powders is challenging due to their low molecular weight sugars and acids, causing stickiness at high temperatures [6,7]. Foam mat drying offers several advantages for processing heat-sensitive foods like fruit juices. It enables rapid drying at lower temperatures, reducing microbial growth and degradation reactions. Benefits include low cost, energy efficiency, short drying time, and value addition [7]. The increased surface area and foam porosity improve mass transfer, further reducing drying time and enhancing product quality [8]. Overall, foam mat drying is a suitable technique for preserving and adding value to perishable foods while retaining their desirable qualities.

Microwave drying is efficient for food preservation, offering benefits like improved storage stability, reduced packaging, and decreased transport weight [5]. Yet, it poses challenges for drying sugar-rich foods like fruit pulps and juices due to their low glass transition temperature, resulting in stickiness at high temperatures [9]. In contrast, foam-mat drying presents a promising alternative for processing aqueous foods, facilitating rapid drying by establishing a stable foam structure that amplifies surface area and diminishes drying time [10]. This technique not only improves texture and rehydration properties but also maintains the foamed layer's thickness, preventing bubble rupture. Notably, heat-sensitive products like fruit juices have shown resilience to microbial and biochemical deterioration when dried using foam-mat techniques, offering increased throughput compared to traditional drying methods [11]. Various factors, including seed components, soluble solids, pulp proportion, and foaming agent concentration, play pivotal roles in influencing foam development and consistency during drying [12]. In essence, while microwave drying provides rapid drying capabilities through electromagnetic wave-induced dielectric heating, foam-mat drying offers unique advantages for preserving heat-sensitive foods with enhanced quality attributes and throughput efficiency.

The intense bitterness found in Nagpur mandarin juice poses a significant challenge in the global processing industry, as it diminishes the commercial value and overall quality of the product. This pervasive bitterness, attributed to flavonoids and limonoids, significantly impacts consumer acceptability and presents a formidable obstacle for the citrus industry at large. Consequently, the processing of Nagpur mandarin fruit and juice has been somewhat overlooked. Hence, the present study is directed towards addressing this critical issue by focusing on the optimization of microwave power, foaming agent utilization, and drying bed thickness during the drying process of Nagpur mandarin juice. Through this investigation, we aim to mitigate the bitterness dilemma and unlock the full potential of Nagpur mandarin in the global citrus market, thereby enhancing its economic viability and consumer appeal.

## 2 Material and methods

### 2.1 Sample preparation

Fully ripe Nagpur mandarin fruit was taken, peeled, and weighed on the weighing balance. The peeled fruit was then used for juice extraction. The juice was squeezed with a juicer (a Bajaj JEX 16 800-Watt Juicer). Fresh juice was blanched at 95°C for 1 min to inactivate the enzyme and cooled quickly [13]. The blanched juice was used for further processing. The foaming agents were added to the juice in the proportions of soy protein isolate (2.10 %, Glycerol mono stearate (GMS) (2.75 %), Carboxyl Methyl Cellulose (CMC) (1.75 %), and sugar (5.10 %), and whipped for 8 min for the formation of foam [14]. Similarly, another sample was prepared with foaming agents guar gum (0.45 %), soy protein isolate (3.30 %), and sugar (10 %) and whipped for 6 min. After whipping the foamed sample, it was uniformly spread over a plate lined with Teflon, and a drying process was performed at 180, 360, 540, 720, and 900 W with bed thicknesses 2, 4, and 6 mm in microwave drying.

### 2.2 Methods for determination of dependent parameters

Drying is the most predominant factor that affects the quality; therefore, the quality of dried Nagpur mandarin juice was evaluated on the basis of various quality parameters like water activity, acidity, ascorbic acid, color, overall acceptability, and drying time. All tests were repeated three times, and the average readings were recorded.

#### 2.2.1 Water activity

The moisture content of food and temperature are the main factors on which water activity depends [15]. Bound water in molecules was defined the water activity. The powder was filled one-third of the volume of the cup provided with the instrument. The instrument took about 1–5 min to display the water activity of the sample. Water activity was measured using a digital water activity meter (AquaLab make model LITE Decagon Devices, Inc. Pullman, Washington DC, USA) for analysis [16].

#### 2.2.2 Acidity

The acidity of the samples was determined by diluting an aliquot of the sample with distilled water and titrating with 0.1 N NaOH using phenolphthalein as an indicator [17]. The calculated acidity was expressed as a percent of anhydrous citric acid.

#### 2.2.3 Ascorbic acid

The ascorbic acid content of the powder was estimated by the titration method [15] using a 2, 6-dichlorophenol indophenol dye solution.

#### 2.2.4 Color kinetics

The change in color of Nagpur mandarin powder was measured by using a colorimeter (CR-20, Konica Minolta, Inc., Japan). The color scale was expressed in L*, a*, and b* by using CIELAB color space [18,19]. L* indicates brightness or darkness in the range of 0–100, and a* represents redness (+) and greenness (−), and b* denotes yellowness (+) and blueness (−) [20]. The color value for each sample was measured for eight replicates. In addition, the total color difference (ΔE*_ab_) and the chroma (C*_ab_) were derived from the measured color values according to Eqs (1) [21].

$$\Delta E=\sqrt{{\left(L_{o}-L_{1}\right)}^{2}+{\left(a_{o}-a_{1}\right)}^{2}+{\left(b_{o}-b_{1}\right)}^{2}}$$

where *L*_0_*, *a*_0_* and *b*_0_* are the color values of fresh Nagpur mandarin juice and *L*_*1*_, *a*_*1*_ and *b*_*1*_ are the color values of Nagpur mandarin powder.

#### 2.2.5 Sensory evaluation

The sensory evaluation was done on the basis of a numerical sensory card based on BIS 6273 (Part II, 1971), as given in Table 1. The Nagpur mandarin powder should have a typical taste, flavor, color, and texture. To test these organoleptic characteristics, sensory evaluation was carried out with the help of a taste panel consisting of 20 panelists. Twenty judges from the faculty and research scholars of the Department of Agricultural Process Engineering, Dr. PDKV, Akola (8 males and 12 females in age range of 25–45) were chosen for the sensory appraisal of the final product (Table 1), focused on good fitness, combined awareness and sensory preferences, capacity to focus and understand, and experience. In keeping with the chosen color, taste, texture and overall acceptability (OAA) attributes for each sample. The sensory evaluation was done on the basis of numerical sensory card based on [22] BIS: 6273 (Part II, 1971) as given in Table 1. The sensory evaluation was carried out for taste and overall acceptability. The samples of Nagpur mandarin powder were served for the evaluation of above parameters to twenty panellists. The score sheet was provided with the product and the panellists were requested to mark the product score according to their liking. Based on the individual marking the average score was computed.

**Table 1:** Score card for sensory evaluation.

Quality grade description	Score
Excellent	8–10
Good	6–7
Fair	4–5
Poor	2–3
Very poor	0–1

#### 2.2.6 Drying time

The time required for drying the foamed Nagpur mandarin juice at different powers and thicknesses was measured until the constant weight was reached.

### 2.3 Optimization of drying parameters

The factorial method deals with a problem of seeking the optimal conditions of the experiment, *i.e*., *the* most desirable. The optimal custom (2FI) design for three variables, i.e., samples (S_1_ and S_2_), microwave power (180, 360, 540, 720, and 900 W), and thickness of bed (2, 4, and 6 mm), including 22 trials formed with 3 replications, was used. This design was selected as it fulfilled most of the requirements needed for optimization of the drying conditions. The experimental design of independent parameters and layout are presented in Table 1 for these three variables, in which there are two levels of sample, five levels of microwave power, and three levels of drying bed thickness under the factorial design.

The goals for factors and responses were carefully selected. For factors, the options included maximizing, minimizing, targeting, keeping within a specified range, or having no specific goal (only applicable to responses). All independent factors (D and T) were maintained within their designated ranges. The responses, including water activity, Del E, and drying time, were minimized, while maximizing for ascorbic acid and overall acceptability. Additionally, the color B value and acidity of all samples were controlled to remain within the desired ranges.

### 2.4 Statistical analyses

The study employed a two-factor randomized complete block design (RCBD) with three replications. Analysis of variance (ANOVA) was conducted on experimental data to assess the main effects of dependent parameters. Experimental values of independent variables were incorporated into a second-order regression model to predict various responses. Using Design Expert 11.0.4.1 statistical software, an optimal custom design was employed to optimize two numerical factors (microwave power and drying bed thickness) and one categorical factor (foaming agent). The Pearson correlation analysis was conducted to determine the relationships between the microwave power level and drying bed thickness on various quality parameters of the powder that were tested.

## 3 Result

### 3.1 Optimization of process parameters for quality enhancement of foamed Nagpur Mandarin juice powder

The optimal custom design was adopted for analysis. The influence of process parameters on quality parameters of powder, viz., acidity, ascorbic acid, water activity, color b value, change in color, overall acceptability, and drying time, were analyzed using the ANOVA technique all results shown in the table (Table 3, Table 4). This table presents experimental runs conducted to analyze the effects of different process parameters on various quality parameters of the powder. These experimental results are then analyzed using statistical techniques to understand the relationships between process parameters and quality parameters, facilitating the optimization of the drying process for desired powder quality.

**Table 2:** Levels of independent variables for microwave drying of Nagpur mandarin juice.

Independent variables	Symbols		Levels	
	Coded	Un-coded	Coded	Un-coded
Sample	A	S	Level 1 of A	S_1_
			Level 2 of A	S_2_
Microwave power	B	P	Level 1 of B	180 W
			Level 2 of B	360 W
			Level 3 of B	540 W
			Level 4 of B	720 W
			Level 5 of B	900 W
Thickness of drying bed	C	T	Level 1 of C	2 mm
			Level 2 of C	4 mm
			Level 3 of C	6 mm

**Table 3:** Dependant parameters of Nagpur mandarin powder at varying process parameters.

Run	MP, W	DBT, mm	FA	WA	Acidity %	AA mg/100 g	Color b value	ΔE	OA	DT, min
1	540	2	S2	0.60	0.26	28.52	17.21	4.70	5.00	46.00
2	360	6	S2	0.59	0.20	27.69	18.59	7.82	5.90	73.00
3	180	4	S2	0.69	0.22	26.26	15.41	10.70	6.20	78.00
4	180	6	S1	0.30	0.84	29.26	18.36	13.01	6.90	58.00
5	180	4	S2	0.65	0.20	24.59	14.65	9.12	6.00	81.00
6	180	2	S1	0.26	0.86	33.25	16.05	11.25	7.00	43.00
7	720	6	S2	0.60	0.32	24.65	17.00	5.21	4.80	47.00
8	540	2	S2	0.59	0.26	25.55	15.72	4.14	5.60	49.00
9	540	4	S1	0.27	0.32	35.21	19.35	6.52	8.00	29.00
10	900	4	S2	0.37	0.14	20.65	15.96	8.07	4.50	36.00
11	540	4	S1	0.26	0.42	37.21	18.63	6.92	7.20	30.00
12	540	4	S2	0.54	0.28	28.25	18.91	4.91	5.80	52.00
13	900	6	S1	0.36	0.60	21.89	11.12	10.62	7.40	25.00
14	900	4	S2	0.40	0.25	19.96	15.97	8.29	4.70	36.00
15	540	2	S1	0.27	0.40	36.98	21.25	6.02	7.60	26.00
16	540	4	S1	0.24	0.49	38.18	18.19	5.98	7.80	28.00
17	540	6	S1	0.30	0.55	38.84	17.66	4.19	8.10	31.00
18	540	4	S1	0.25	0.37	37.14	20.49	4.90	8.30	31.00
19	720	6	S2	0.48	0.43	25.55	15.94	4.02	4.50	47.00
20	900	2	S2	0.47	0.19	18.99	14.17	9.02	4.30	27.00
21	900	2	S1	0.23	0.22	21.76	17.48	12.64	7.50	18.00
22	180	2	S2	0.64	0.22	22.54	13.57	8.79	6.50	75.00

Note: MP represents Microwave Power (W), DBT denotes Drying Bed Thickness (mm), FA indicates the Foaming Agent used, WA signifies Water Activity, AA represents Ascorbic Acid concentration (mg/100 g), ΔE signifies the Change in Color, OA denotes Overall Acceptability, and DT represents Drying Time (min). Foaming agents were categorized into two types: S1 (consisting of Guar gum (0.45 %), soy protein isolate (3.30 %), and sugar (10 %) with whipping times of 6 min) and S2 (involving foamed juice produced by soy protein isolate (2.10 %), GMS (2.75 %), CMC (1.75 %), and sugar (5.10 %) with whipping times of 8 min).

**Table 4:** The anova and Regression coefficients, standard deviation (Std. Dev.), R^2^, and CV values for the effect of microwave power, foaming agents, and drying bed thickness on seven dependent variables of Nagpur mandarin powder.

Source	Water Activity	Acidity	Ascorbic Acid	b value	ΔE	Overall acceptability	Drying Time
Model	403.73^a^	18.52^a^	57.90^a^	15.05^a^	45.09^a^	35.22^a^	214.81^a^
A	41.08^a^	13.30^a^	42.40^a^	6.37*	6.24*	8.96*	811.28*
B	8.56^a^	8.54	–	–	–	–	80.44^a^
C	1407.90^a^	63.00^a^	93.36^a^	5.99*	23.10^a^	201.27^a^	624.50^a^
A^2^	33.78^a^	4.14*	101.29^a^	36.65^a^	155.65^a^	4.04*	35.76^a^
B^2^	–	9.65^a^	5.96*	–	–	–	–
C^2^	–	–	–	–	–	–	–
AB	–	5.49*		16.90^a^	8.23*	–	
AC	–	35.97^a^	4.93	–	–	27.06^a^	42.37^a^
BC	–	–	–	12.81^a^	–	–	–
Lack of Fit	1.27^NS^	3.24 ^NS^	2.51 ^NS^	2.03 ^NS^	1.54 ^NS^	0.93 ^NS^	3.48 ^NS^
Std.Dev	0.02	0.08	1.71	1.05	0.91	0.36	2.07
C·V (%)	4.52	5.53	6.04	6.23	4.06	5.61	4.72
R^2^	0.9896	0.9025	0.9476	0.8575	0.9139	0.9559	0.9925
Adj R^2^	0.9871	0.8538	0.9313	0.8005	0.8936	0.9288	0.9879
Pred R^2^	0.9827	0.6616	0.8939	0.6480	0.8528	0.8710	0.9625

a is 1 % level of significance * is 5 % level of significance and NS is non significance.

### 3.2 Water activity

The water activity is the most important parameter for producing a shelf-stable product and is to be minimized during processing. The water activity of Nagpur mandarin powder ranged from 0.23 to 0.69 (Table 3). The maximum water activity was found to be 0.69 for 180 W microwave power and 4 mm thickness with a foaming agents guar gum (0.45 %), soy protein isolate (3.30 %), and sugar (10 %) and whipped for 6 min, while the minimum water activity was found to be 0.23 for 900 W microwave power and 2 mm thickness and foam formed with soy protein isolate (2.10 %), GMS (2.75 %), CMC (1.75 %), and sugar (5.10 %) with whipping times of 8 min. In our study the minimum water activity was found with the maximum microwave power, and vice versa.

**Fig. 1:**
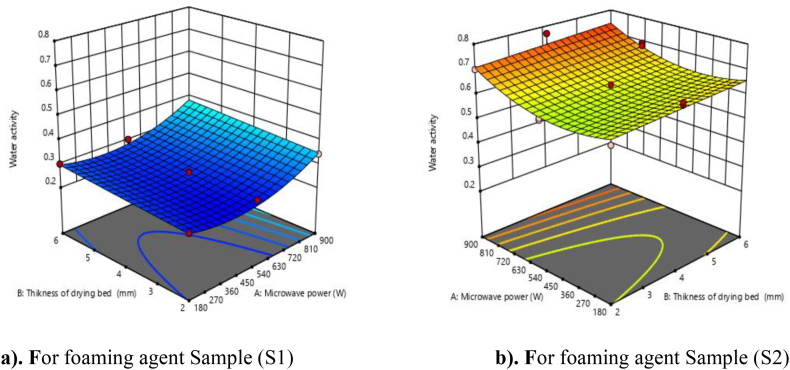
**(a, b).** Response surface plots (3D) for water activity as function of foaming agents, microwave power and drying bed thickness.

It was found that the effects of microwave power, foaming agent, and drying bed thickness were significant at the 1 % level of significance. The water activity increases with an increase in drying bed thickness and foaming agent and decreases with an increase in microwave power similar results were found in correlation analysis. From Table 4, the quadratic model was found to be best fitted to the experimental data and to be significant for linear and quadratic terms for water activity. The R^2^ value was found to be 0.9896, which shows a good fit of the model with an F value of 403.73 (P < 0.01). The linear terms (A, B, and C) and quadratic terms (A2) were significant, while the interactive terms (AB, AC, and BC) and quadratic terms (B^2^ and C^2^) were found non-significant. The lack of fit F value was non-significant, which indicates that the developed model was adequate for predicting the response. Moreover, the predicted R^2^ of 0.987 was in reasonable agreement with the adjusted R^2^ of 0.9827. To visualize the combined effect of process variables on water activity, the response surface and contour plots were fitted as a function of two variables while keeping the third variables at their central values. The effect of microwave power, drying bed thickness, and foaming agent on water activity were presented in Fig. 1(a and b), it shows that the microwave power increases the water activity decreases whereas drying bed thickness increases the water activity also decreases.

### 3.3 Acidity

It was found that the effects of microwave power and foaming agent were significant on acidity at the 1 % level of significance while drying bed thickness was significant at 5 %. The quadratic model was found to be best fitted to the experimental data and was found significant for linear, quadratic, and interaction terms, which were calculated for acidity and shown in Table 4. The R^2^ value was determined by the least-squares technique and found to be 0.9025, showing a good fit of the model with an F value of 18.52 (P < 0.01). The P-values less than 0.05 indicate that the model terms are significant. In this case, linear terms (A, B, and C), interactive terms (AB and AC), and quadratic terms (A2 and B2) were significant model terms. The effect of the interactive term (BC) and quadratic term (C2) was found to be non-significant. The lack of fit F-value (3.24) implies that the lack of Fit was not found to be significant relative to the pure error. The predicted R^2^ (0.6616) was in reasonable agreement with the adjusted R^2^ (0.8538).

**Fig. 2:**
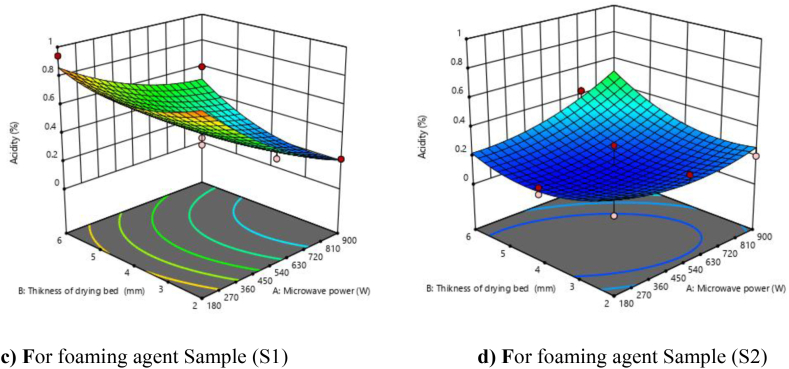
**(c, d).** Response surface plots (3D) for acidity as function of foaming agents, microwave power and drying bed thickness.

To visualize the combined effect of three variables on the acidity, the response surface and contour plots were fitted as a function of two variables while keeping the third variable at its central value. From Fig. 2(c and d), it was found that the acidity increases with a decrease in microwave power and increases with an increase in drying bed thickness.

### 3.4 Ascorbic acid

The maximum ascorbic acid was found at 38.84 mg/100 g at 540 W microwave power, 6 mm thickness in the foaming agents soy protein isolate (2.10 %), GMS (2.75 %), CMC (1.75 %), and sugar (5.10 %) with whipping times of 8 min, while the minimum ascorbic acid was found at 18.99 mg/100 g at 900 W microwave power, 2 mm thickness in the sample 2 (guar gum (0.45 %), soy protein isolate (3.30 %), and sugar (10 %) and whipped for 6 min) (Table 3). The samples and foam were formed with soy protein isolate (2.10 %), GMS (2.75 %), CMC (1.75 %), and sugar (5.10 %) with whipping times of 8 min Retained more ascorbic acid as compared to samples with foaming agent guar gum (0.45 %), soy protein isolate (3.30 %), and sugar (10 %) and whipped for 6 min. The increase in vitamin C content was due to the hydrocolloid activity of methylcellulose on vitamin C during drying.

**Fig. 3:**
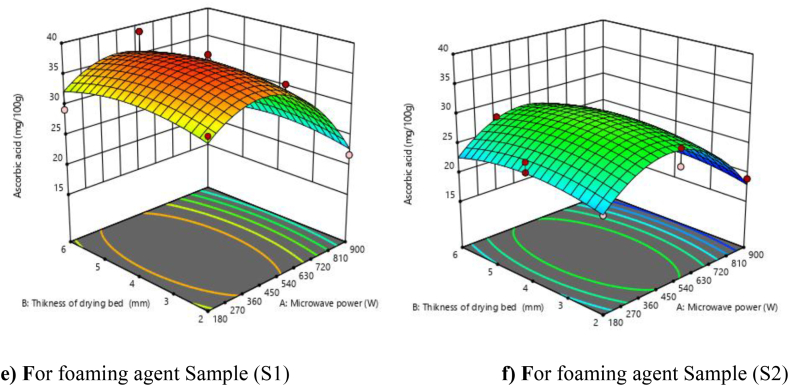
**(e, f).** Response surface plots (3D) for ascorbic acid as function of foaming agents, microwave power and drying bed thickness.

The model fitted to ascorbic acid was found significant at the 1 % level in linear terms of microwave power and foaming agent. From Fig. 3 (e and f), it shows that the ascorbic acid was increased with an increase in microwave power up to 540 W, and afterwards, decreasing trends were observed. This result was due to the destructive effect of prolonged thermal treatment, which caused the oxidation of ascorbic acid during drying.

The quadratic model was found to be best fitted to the experimental data, which was significant for linear, quadratic, and interaction terms (Table 4). The R2 value was calculated by the least-squares technique and found to be 0.9476, showing a good fit of the model. An F value of 57.90 implies that the model is significant (P < 0.01). The linear terms (A and C), the interactive term (AC), and quadratic terms (A^2^ and B2) were significant, while the linear term (B), interactive terms (AB and BC), and quadratic term (C^2^) were found non-significant. The lack of fit F value of 2.51 implies that the lack of fit was not significant relative to the pure error. Moreover, the predicted R^2^ (0.8939) was in reasonable agreement with the adjusted R^2^ (0.9313).

### 3.5 Color (b-value)

**Fig. 4:**
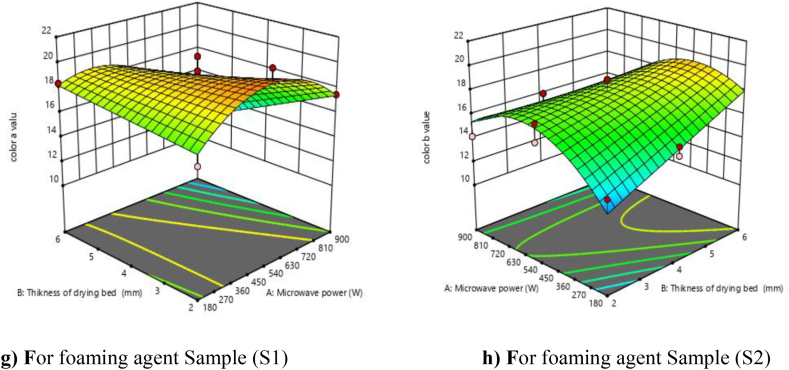
**(g, h).** Response surface plots (3D) for b value as function of foaming agents, microwave power and drying bed thickness.

Color is often used as an indication of quality and freshness for food products. Hence, it has become important for food processors to evaluate and grade their products based on color. The color of mandarin powder was measured in terms of b-value (brightness or yellowness). It was observed from Fig. 4 (g and h) that the b value increased with an increase in microwave power from 180 to 540 W and then decreased, while the b value increased with an increase in drying bed thickness. Similar results we can be seen in correlation analysis. The quadratic model was found to be best fitted to the experimental data, which was significant for linear, quadratic, and interaction terms as shown in Table 4. The R^2^ value was calculated by the least-squares technique and was found to be 0.8575, which shows the good fit of the model. The model F value was 15.05, which was found to be significant (P < 0.01). The linear terms (A and C), interaction terms (AB and BC), and quadratic terms (A2) were found significant, while the linear term (B), the interactive term (AC), and the quadratic term (B2 and C2) were found non-significant. The lack of fit in the F value (2.03) was non-significant. The predicted R2 (0.648) was found to be in reasonable agreement with the adjusted R2 (0.8005).

### 3.6 Color (ΔE value)

**Fig. 5:**
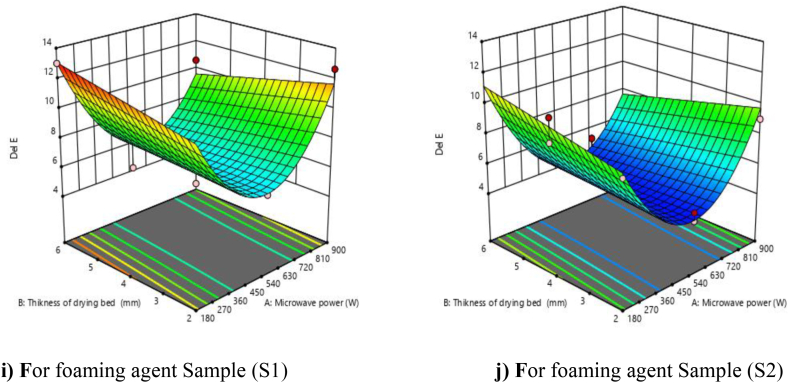
**(ij).** Response surface plots (3D) for ΔE value as function of foaming agents, microwave power and drying bed thickness.

The ΔE value represents the change in color of the product, which is used as a quality indication of the food product. Hence, it has become important for food processors to evaluate the change in color. The change in color of mandarin powder was measured in terms of the ΔE value, as shown in Table 3. From Fig. 5 (i and j), it shows that the color ΔE value decreased with an increase in microwave power from 180 to 540 W afterwards, and a non-significant effect was found with drying bed thickness on the color ΔE value. The microwave drying process does not cause the phenomenon of surface overheating, resulting in less change in the surface color. The lack of fit F value was non-significant, which indicates that the developed model was adequate for predicting the response. Moreover, the predicted R^2^ (0.8528) was in reasonable agreement with the adjusted R^2^ (0.8936). The R^2^ (0.9139) and F (45.09) values indicate that the model was significant (P < 0.01). In this case, linear terms (A and C), interaction terms (AB), and quadratic terms (A^2^) were found to be significant. The effects of the linear term (B), interactive terms (BC and AC), and quadratic terms (B^2^ and C^2^) were found to be non-significant.

### 3.7 Overall acceptability

The maximum overall acceptability (8.30) was found at 540 W microwave power and 4 mm thickness, and foam formed with soy protein isolate (2.10 %), GMS (2.75 %), CMC (1.75 %), and sugar (5.10 %) with whipping times of 8 min. While minimum overall acceptability (4.30) was found at 900 W microwave power and 2 mm thickness in a foaming agents guar gum (0.45 %), soy protein isolate (3.30 %), and sugar (10 %) and whipped for 6 min (Table 3), The sample foamed using soy protein isolate (2.10 %), GMS (2.75 %), CMC (1.75 %), and sugar (5.10 %) with whipping times of 8 min and had the highest overall acceptability as compared to a Sample 2 (foaming agents guar gum (0.45 %), soy protein isolate (3.30 %), and sugar (10 %) and whipped for 6 min). It was observed that the effect of microwave power was significant on overall acceptability at the 1 % level of significance, and the foaming agent was found significant at 5 %.

**Fig. 6:**
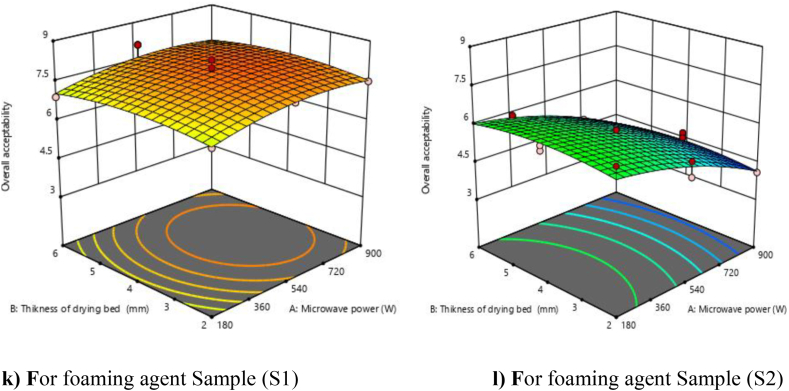
**(k, l).** Response surface plots (3D) for overall acceptability as function of foaming agents, microwave power and drying bed thickness.

The R^2^ value was calculated and found to be 0.9559, which shows the good fit of the model with an F value of 35.22, which implies that the model was significant (P < 0.01). In this case, linear terms (A and C), interactive terms (AC), and quadratic terms (A^2^) were found significant, while linear terms (B), interactive terms (AB and BC), and quadratic terms (B^2^ and C^2^) were found non-significant. The lack of fit F-value (0.93) was found to be not significant relative to the pure error, which indicates that the developed model was adequate for predicting the response. The predicted R^2^ (0.8710) was in reasonable agreement with the adjusted R^2^ (0.9288). The combined effect of all variables on the overall acceptability, the response surface and contour plots (Fig. 6 k and l) and it shows that the overall acceptability of powder was increases with increase in microwave power from 180 to 540 W, afterwards the trend was found decreasing with increase in microwave power (540–900 W).

### 3.8 Drying time

**Fig. 7:**
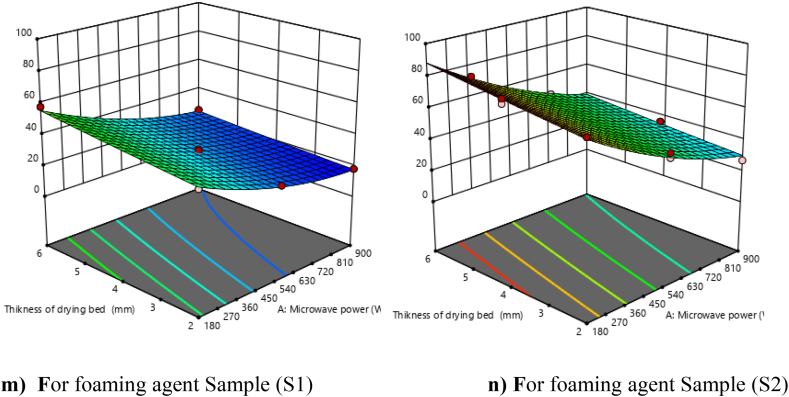
**(m, n).** Response surface plots (3D) for drying time as function of foaming agents, microwave power and drying bed thickness.

The initial moisture content of foamed Nagpur mandarin juice was found to be 89.35 % and 87.59 % (wb) with an S1 and S2 foaming agent, and the final moisture content was found to be 1.40–4.70 % (wb). The drying time of mandarin powder was found to range from 18.00 to 81.00 min at varying microwave powers of 180–900 W (Table 3). In Fig. 7 (m and n), it is revealed that microwave power is the most predominant factor in drying time. As microwave power increases, the drying time decreases. It was also observed that the drying time increased with increasing drying bed thickness.

It was observed that the effects of microwave power, drying bed thickness, and foaming agent were significant on drying time at the 1 % level of significance. The R^2^ value was calculated and found to be 0.9925, which shows the good fit of the model, with an F value of 214.81, which implies that the model was significant (P < 0.01). It reveals that linear terms (A, B, and C), the interactive term (AC), and the quadratic term (A^2^) were significant. The interactive terms (AB and BC) and quadratic terms (B^2^ and C^2^) were found to be non-significant. The lack of fit F-value (3.48) was found not to be significant. Moreover, the predicted R^2^ (0.9625) was in reasonable agreement with the adjusted R^2^ (0.9879), revealing that the non-significant terms were not included in the model.

### 3.9 Optimal optimization of microwave power, drying bed thickness, and foaming agent for the drying process of foamed Nagpur Mandarin juice

The optimum range of process parameters was presented in Table 5 with importance. To optimize the process parameters for preparation of powder by categorical factor optimization; which finds a point that maximizes the desirability function. The optimum operating conditions for drying foamed juice in microwave drying (microwave power, drying bed thickness, and foaming agent) are presented in Table 5.

**Table 5:** Constraints for optimization of microwave power, foaming agent and drying bed thickness of Nagpur mandarin juice.

Name	Goal	Lower Limit	Upper Limit	Lower Weight	Upper Weight	Importance
A: Microwave power, W	is in range	180	900	1	1	3
B: Thickness of drying bed, mm	is in range	2.00	6.00	1	1	3
C: Sample	is in range	AFA	NFA	1	1	3
Water activity	Minimize	0.24	0.77	1	1	3
Acidity, %	is in range	0.12	0.89	1	1	3
Ascorbic acid, mg/100 g	Maximize	18.99	38.84	1	1	3
color b value	Maximize	11.12	21.25	1	1	3
change in color (ΔE)	Minimize	4.025	13.01	1	1	3
Overall acceptability	Maximize	4.10	8.30	1	1	3
Drying time, min	Minimize	18.00	81.00	1	1	3

Note: AFA is an artificial foaming agent and NFA is the natural foaming agent.

**Fig. 8:**
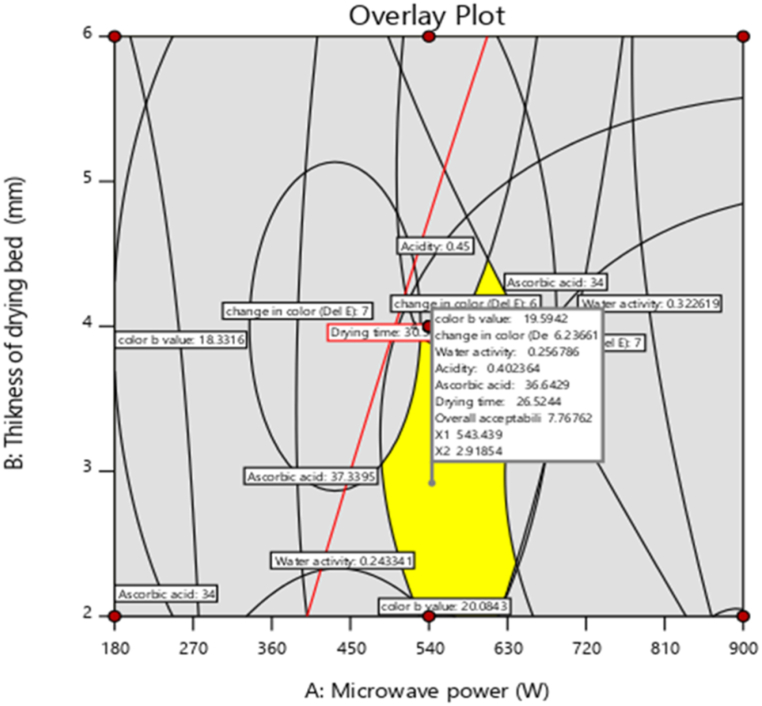
Superimposed Contour plots for optimization of microwave power, drying bed thickness and foaming agent for development of powder.

A graphical multi-response optimization technique was adopted to determine the workable optimum conditions for the development of Nagpur mandarin powder. The contour plots for all responses were superimposed and regions that best satisfy all the constraints were selected as optimum conditions. These constraints resulted in a ‘feasible zone’ of the optimum conditions (shaded area in the superimposed contour plots). The superimposed contour plots having a common superimposed area for all responses were shown in Fig. 8.

Based on the criteria as given in Table 5, the optimization was carried out. During optimization, the solutions were obtained, out of which one best-suited solution was selected and as shown in Table 6.Where, MP is microwave power, DBT is drying bed thickness, S is sample, WA is water activity, AA is water activity, ΔE is change in color, OA is overall acceptability, DT is drying time, D is desirability and S2 foamed juice produced by soy protein isolate (2.10 %), GMS (2.75 %), CMC (1.75 %), and sugar (5.10 %) with whipping times of 8 min.

**Table 6:** Solution for optimization of microwave power, drying bed thickness, foaming agent for development of powder.

MP,W	DBT,mm	S	WA	Acidity %	AA mg/100 g	Color b value	ΔE	OA	DT, min	D
543.44	2.92	S2	0.26	0.40	36.64	19.59	6.24	7.77	26.52	0.84

The optimal values for the process variables obtained through categorical optimization are: Foaming agent (F): Artificial, Microwave power (MP): 540 W, Thickness of drying bed (Td): 3 mm. The ideal conditions for the production of Nagpur mandarin powder were identified as using a microwave power of 540 W for drying, with a drying bed thickness of 3 mm and artificial foaming agent. Under these optimized conditions, the quality parameters were assessed as follows: color b value of 19.59, ΔE (change in color) of 6.24, acidity of 0.40 %, ascorbic acid content of 36.64 mg/100 g, water activity of 0.26, drying time of 26.52 min, and overall acceptability score of 7.77. The desirability of the optimized solution was determined to be 0.84 (Table 6).

#### 3.9.1 Model validation for Nagpur Mandarin powder development

An experiment was conducted to verify the adequacy of the drying experiment in predicting response values under optimal process conditions. Table 7 presents both experimental and predicted values, derived from three experiments. The results showed close similarity between experimental and predicted values, leading to the conclusion that the model effectively assesses drying behaviour.

**Table 7:** Predicted and experimental values of response at optimum process conditions for drying of Nagpur mandarin juice.

Response	Predicted mean	Experimental mean Value ± SD	SE	% Variation	Mean difference
Water activity	0.26	0.26 ± 0.02	0.01	0.05	0.01
Acidity, %	0.40	0.412 ± 0.08	0.04	0.16	0.01
Ascorbic acid, mg/100 g	36.64	32.14 ± 1.71	0.66	2.79	4.50
Color b value	19.59	18.38 ± 1.05	0.41	1.75	1.21
Del E	6.24	5.69 ± 0.93	0.41	1.78	0.55
Overall acceptability	7.77	7.49 ± 0.36	0.16	0.68	0.28
Drying time, min	26.52	28.00 ± 1.21	0.54	2.32	1.48

### 3.10 Correlation analysis of microwave power (MP) and drying bed thickness (DBT) for the quality parameter

**Fig. 9:**
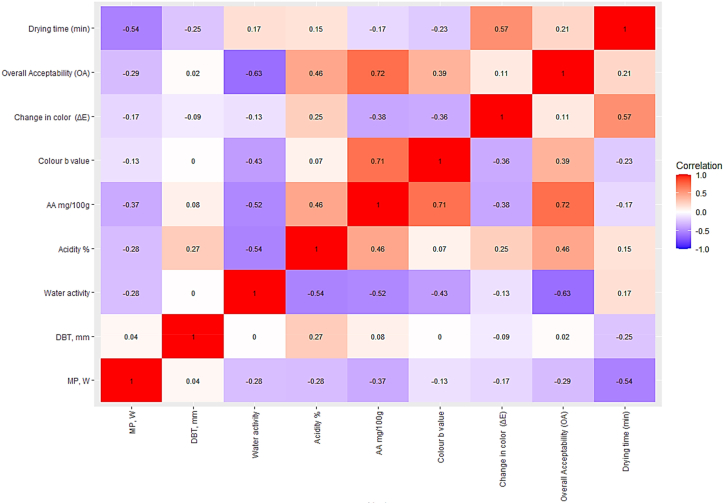
Correlation between microwave power (MP) and various parameters.

The correlation coefficient is a statistical measure often used in studies to show an association between variables or to look at the agreement between two methods. This correlation Fig. 9 presents the relationships between various parameters measured in a study. MP exhibits a moderate negative correlation with water activity (correlation coefficient = −0.2791), indicating that higher microwave power levels may lead to lower water activity. Additionally, MP shows a moderate negative correlation with overall acceptability (correlation coefficient = −0.2879), suggesting that higher microwave power levels might be associated with decreased overall acceptability. However, these correlations are not very strong, implying that other factors may also influence water activity and overall acceptability independently of microwave power. MP also displays a moderate negative correlation with drying time (correlation coefficient = −0.5439), indicating that higher microwave power levels may be associated with shorter drying times.

The correlation between DBT (Drying Bed Thickness) measured in millimeters and other parameters reveals interesting relationships. DBT, mm exhibits a weak positive correlation with acidity percentage (correlation coefficient = 0.2697), suggesting a slight tendency for higher DBT values to be associated with higher acidity levels. However, this correlation is not very strong, indicating that other factors may also influence acidity levels independently of DBT. Notably, DBT, mm displays a moderate negative correlation with color b value (correlation coefficient = −0.2513), indicating that thicker drying bed thickness may be associated with lower color values. Additionally, DBT, mm shows very weak positive correlations with overall acceptability (correlation coefficient = 0.0208) and water activity (correlation coefficient = 0.0035), implying minimal associations between these variables and drying bed thickness. The correlations between DBT, mm, and other parameters are relatively low, suggesting that DBT may not be strongly indicative of variations in these specific factors.

## 4 Discussion

Foam mat drying is an intriguing advancement in the field of aqueous foodstuffs, in which a fluid or semi-liquid is thermally dehydrated into a stable foam. Compared to other drying techniques, such as vacuum, freeze, and spray drying, foam drying is less expensive, retains nutritional qualities, and yields as a powder at a lower cost [7,23,24]. Its prevalence in processing fruit pulp and juices, vegetable and shrimp purees, fruit extracts, and even seed extracts makes it a reliable technique, especially for sticky, high-sugar materials that usually cause problems using other drying techniques. Microwave heating is considered the most efficient source for drying, offering excellent heat conduction to the dried material's interior, cleanliness, energy recovery, quick start and stop of the drying process, and energy process management [20,[25], [26], [27]].

### 4.1 The evaluation of quality attributes of foam mat drying process and product quality

The water activity (aw) of Nagpur mandarin powder obtained in this study ranged from 0.24 to 0.69 (Table 2). Foods with a water activity of less than 0.6 are considered microbiologically stable [28]. It indicates that any degradation that occurs in the powder is more likely caused by chemical processes rather than microorganisms. Inhibiting oxidative and enzymatic processes is crucial for maintaining the quality of food products [29] and reducing water activity serves this purpose.

Interestingly, in our study, the guar gum foaming agent exhibited higher water activity compared to the GMS (glycerol monostearate) and CMC (carboxymethyl cellulose) foaming agents. This observation might be attributed to the structure of guar gum. Guar seed endosperm, a source of water-soluble gum, contributes to the soluble dietary fiber (SDF) component of the seed's total dietary fiber (TDF). Guar gum is widely used as a thickener, emulsifier, and stabilizer in various food items [[30], [31]]. It serves multiple functions as a food additive, including emulsification, water binding, stabilization, prevention of ice crystal formation in frozen goods, moisturizing, thickening, and suspension of liquid-solid systems [32,33]. Typically, guar gum is used in ice cream, sauces, cake mixes, cheese spreads, fruit drinks, and dressings in amounts less than 1 % of the product weight. The variation in water activity points to the potential impact of different foaming agents on the overall quality and stability of the food product.

In the present study, the acidity value of the samples ranged from 0.86 to 0.19 %. This variation can be attributed to the sugar content in the foaming treatments. Sugar has been known to increase sweetness while reducing sourness, leading to a decrease in acidity percentages. Interestingly, the acidity content varied at different microwave power levels during the drying process. Increased microwave power led to decreased acidity levels compared to samples processed at lower power, consistent with our correlation analysis. This trend aligns with findings from other studies regarding free fatty acid (FFA) levels. For instance Ref. [34], observed decreasing FFA values with higher microwave power and temperature in brown shrimps. Additionally [35], reported lower FFA levels in microwave-dried tilapia compared to sun-dried tilapia, suggesting the inactivation of lipases and phospholipases during microwave drying. This trend is further supported by the observation of lower FFA levels in microwave-dried carp fillets, possibly due to lipase inactivation or the loss of volatile FFAs. The influence of microwave power on the acidity content of food products during drying warrants further investigation.

The study found that different drying and foaming conditions significantly impacted the amount of ascorbic acid, a critical antioxidant, with levels ranging from 18.99 to 38.84 mg/100 g (as shown in Table 3). Ascorbic acid plays an essential role in a balanced diet and is naturally present in various plant-based foods. However, its degradation during food processing and preservation is a primary concern, especially with heat exposure. Traditional preservation methods such as freezing or drying typically lead to a decline in ascorbic acid levels. The comparison of microwave power treatments revealed that the duration of drying and the heat source significantly influenced ascorbic acid degradation. Specifically, in our study lower heat supply with longer drying periods resulted in decreased ascorbic acid content, while higher heat supply with shorter drying times contributed to better retention of ascorbic acid. This aligns with the findings of [36], who noted that ascorbic acid is more sensitive to degradation when exposed to prolonged drying durations and low temperatures compared to shorter durations and higher temperatures. Understanding the impact of drying conditions on ascorbic acid content is crucial for maintaining the nutritional quality of food products and optimizing processing techniques.

The study found that the foaming agents present in the mandarin juice foam caused the color of the foam to be lighter than that of the pulp, resulting in an increase in luminosity and a decrease in a* and b* values. However, the heating of fruit sugars stabilizers, and foam-forming agents caused a darkening effect during the drying process. As in our study microwave power levels increased, sensory scores for color, flavor, taste, and overall acceptability decreased significantly, consistent with our correlation analysis. Similar patterns were observed in the sensory quality of foam-mat dried tamarind powders [37,38], banana paste [39], and sea buckthorn (Hippophae salicifolia) leather [37]. The volatile components present in mandarin juice contribute to its unique flavor and aroma, and their loss during the drying process may lead to a decline in sensory quality. The drying time varied significantly based on the foaming and drying conditions implemented, with foaming agents reducing the drying time by promoting faster moisture diffusion and removal. Microwave energy, which efficiently penetrates the material, also promotes rapid moisture movement and evaporation, reducing overall drying time. However, faster drying times may lead to a loss of volatile components and a decline in sensory quality. These findings align with previous research on foam-mat drying of various fruits and vegetables [40], foamed raspberry puree [41], and cassava [42].

### 4.2 Potential implications and future research

The findings of this study offer significant implications for foam mat drying applications in citrus fruit processing, particularly for fruits like Nagpur mandarin. These implications include extended shelf-life attributed to low water activity, enabling prolonged storage and transportation of dried fruit powders, while retaining essential nutrients such as vitamin C (ascorbic acid), enhancing their nutritional value. The concentrated flavor, color, and nutritional properties of these powders make them versatile ingredients for various food industry applications, including beverages, bakery, confectionery, and supplements, thus reducing post-harvest losses and adding value by utilizing surplus or damaged fruits. Moreover, the potential synergy between foaming agents and drying techniques warrants further exploration, such as combining foaming agents with freeze-drying or spray-drying, to enhance efficiency and product quality. These areas for future research hold promise for advancing food-drying technologies, leading to innovative solutions with improved sensory attributes and reduced processing times.

## 5 Conclusions

In conclusion, this study focused on the optimization of microwave power, foaming agent selection, and drying bed thickness to enhance the quality parameters of Nagpur mandarin powder. The optimization of microwave power, foaming agent, and drying bed thickness was done using the optimal custom design. The optimum condition for the development of powder was found to be 540 W microwave power for drying with a drying bed thickness of 3 mm and foaming agents were used in the proportion of soy protein isolate (2.10 %, GMS (2.75 %), CMC (1.75 %), and sugar (5.10 %), and whipped for 8 min. The quality parameters at optimized conditions, viz. Color b value, E, acidity, ascorbic acid, water activity, drying time, and overall acceptability, were found to be 19.59, 6.24, 0.40 %, 36.64 mg/100 g, 0.26, 26.52 min, and 7.77, respectively. The desirability of the optimized solution was found to be 0.84.

## Ethics statement

The research followed recognized ethical standards. Every volunteer on the sensory panel provided written consent before participating, having been briefed on the study's goals and procedures to safeguard their data confidentiality. All participants were in good health and had no known allergies to the tested components.

## Data availability statement

Data will be made available on request.

## CRediT authorship contribution statement

**Bhagyashree Nivrutti Patil:** Writing – original draft, Visualization, Supervision, Software, Methodology, Investigation, Formal analysis, Data curation, Conceptualization. **Suchita V. Gupta:** Writing – review & editing, Validation, Supervision, Methodology, Investigation, Formal analysis. **N.B. Patil:** Writing – original draft, Software, Methodology, Investigation, Formal analysis, Data curation, Conceptualization. **Nileshwari Yewle:** Writing – review & editing, Validation, Supervision, Software, Methodology, Investigation, Formal analysis.

## Declaration of competing interest

The authors declare that they have no known competing financial interests or personal relationships that could have appeared to influence the work reported in this paper.
